# Assessment of phytochemical content, polyphenolic composition, antioxidant and antibacterial activities of Leguminosae medicinal plants in Peninsular Malaysia

**DOI:** 10.1186/1472-6882-11-12

**Published:** 2011-02-10

**Authors:** Yik Ling Chew, Elaine Wan Ling Chan, Pei Ling Tan, Yau Yan Lim, Johnson Stanslas, Joo Kheng Goh

**Affiliations:** 1 School of Science, Monash University Sunway Campus, Bandar Sunway, 46150 Petaling Jaya, Selangor, Malaysia; 2 Pharmacotherapeutics Unit, Department of Medicine, Faculty of Medicine and Health Sciences, Universiti Putra Malaysia, 43400 Serdang, Selangor Darul Ehsan, Malaysia; 3 Laboratory of Natural Products, Institute of Bioscience, Universiti Putra Malaysia, 43400 Serdang, Selangor Darul Ehsan, Malaysia

## Abstract

**Background:**

Many medicinal plants from Leguminosae family can be found easily in Malaysia. These plants have been used as traditional medicines by local ethnic groups, where they are prepared as decoction, pastes for wound infections, and some have been eaten as salad. This paper focused on the assessment of antioxidant potential, antibacterial activity and classes of phytochemicals of nine plants from the Leguminosae family.

**Methods:**

*Acacia auriculiformis*, *Bauhinia kockiana*, *Bauhinia purpurea*, *Caesalpinia pulcherrima*, *Calliandra tergemina*, *Cassia surattensis, Leucaena leucocephala*, *Peltophorum pterocarpum*, and *Samanea saman *were extracted with aqueous methanol and dichloromethane:methanol mixture to test for antioxidant and antibacterial activities. The Folin-Ciocalteu assay was conducted to quantify the total phenolic content and 2, 2-diphenyl-1-picrylhydrazyl (DPPH) assay was used to determine the free radical quenching capacity. Antibacterial activity was assessed using disc diffusion (Kirby-Bauer) assay. Screening for major classes of phytochemical was done using standard chemical tests.

**Results:**

*B. kockiana *flowers and *C. pulcherrima *leaves contained high total phenolic content (TPC) and strong DPPH radical scavenging ability with TPC of 8280 ± 498 mg GAE/100 g, IC_50 _of 27.0 ± 5.0 μg/mL and TPC of 5030 ± 602 mg GAE/100 g, IC_50 _of 50.0 ± 5.0 μg/mL respectively. Positive correlation was observed between TPC and free radical scavenging ability. Most extracts showed antibacterial activity against Gram positive bacteria at 1 mg, while none showed activity against Gram negative bacteria at the same dose. All extracts (except *Samanea saman *flower) showed antibacterial activity against two strains of methicillin resistant *Staphylococcus aureus *(MRSA) with MID values ranging between 100 μg/disc and 500 μg/disc.

**Conclusion:**

The potential source of antioxidant and antibacterial agents, especially for MRSA infection treatments were found in *B. kockiana*, *C. pulcherrima*, *C. tergemina *and *P. pterocarpum*. These preliminary results would be a guide in the selection of potential candidates for further pharmacological study and in search of new drug candidate in treating MRSA infections.

## Background

Natural products and secondary metabolites formed by living systems, notably from plant origin, have shown great potential in treating human diseases such as cancer, coronary heart diseases, diabetes and infectious diseases [1]. According to World Health Organisation, 65 - 80% of the world populations rely on traditional medicine to treat various diseases [2]. To date, many plants have been claimed to pose beneficial health effects such as antioxidant and antimicrobial properties. With the emergence of multiple strains of antibiotic resistance microorganism, great interest has been generated in search for potential compounds from plants for therapeutic, medicinal, aromatic and aesthetic uses [2,3].

Phytochemicals are natural and non-nutritive bioactive compounds produced by plants that act as protective agents against external stress and pathogenic attack [4]. Secondary metabolite is crucial for plant defenses (*e.g. *as an antioxidant or antimicrobial agent) which has enabled plants to survive. Based on their biosynthetic origin, phytochemicals can be divided into several categories: phenolics, alkaloids, steroids, terpenes, saponins, *etc*. Phytochemicals could also exhibit other bioactivities such as antimutagenic, anticarcinogenic, antioxidant, antimicrobial, and anti-inflammatory properties [5]. These plant-derived phytochemicals with therapeutic properties could be used as single therapeutic agent or as combined formulations in drug development.

Phenolic is one of the major groups of phytochemical that can be found ubiquitously in certain plants. Phenolic compounds are potent antioxidants and free radical scavenger which can act as hydrogen donors, reducing agents, metal chelators and singlet oxygen quenchers [4]. Studies have shown that phenolic compounds such as catechin and quercetin were very efficient in stabilising phospholipid bilayers against peroxidation induced by reactive oxygen species (ROS) [6,7].

In Malaysia, only 5 - 15% of more than 250000 species of higher plants with therapeutic potential have been studied [8]. Hence, there is a vast potential to reveal plant resources with useful phytochemicals [9].

The bioactivities of nine medicinal plants from Leguminosae family were assessed in this report. The Leguminosae family comprises of 650 genera and more than 18000 species [9]. Some of these plants were reported as medicinal plants, such as *B. kockiana*, *B. purpurea*, *C. pulcherrima*, *C. surattensis *which have been used traditionally to treat various diseases. Also, these medicinal plants showed various bioactivities such as anti-cancer, anti-inflammatory, antimicrobial and antioxidant properties. For example, *Glycyrrhiza uralensis *(kan cao) root ethanolic extracts has chemopreventive properties where it was found to induce apoptosis in MCF-7 breast cancer cells [10]; *Trigonella foenum-graecum *(fenugreek) seed extract has shown to have hypoglycaemic and hypocholesterolemic properties [11]; isoflavonoids isolated from *Erythrina variegata *significantly inhibit the growth of MRSA [12]; and *Vicia faba *(broad bean) showed antimicrobial activity against bacteria such as *Escherichia coli*, *Shigella *sp., *Bacillus subtilis *and *Staphylococcus aureus *[13].

The main aims of this report are to evaluate antioxidant capacity (total phenolic content and free radical scavenging ability), antibacterial activity and to screen for phytochemicals content in flowers and leaves of nine medicinal plants from Leguminosae family, namely *Acacia auriculiformis*, *Bauhinia kockiana*, *Bauhinia purpurea*, *Caesalpinia pulcherrima*, *Calliandra tergemina*, *Cassia surattensis, Leucaena leucocephala*, *Peltophorum pterocarpum*, and *Samanea saman*. To the best of our knowledge, this is the first report on antioxidant and antibacterial activities of most flowers and leaves of selected Leguminosae species.

## Methods

### Plant materials and preparation of extracts

Flowers and leaves of plants of interest were collected from Klang Valley in Peninsular Malaysia and were identified by plant taxonomist, Mr. Anthonysamy Savarimuthu. The plant materials were collected on the day when extraction was performed. Voucher specimens (MUM-LEGUM-001 - MUM-LEGUM-009) were deposited in the herbarium of School of Science, Monash University Sunway campus.

#### Antioxidant activity

Extraction of flowers and leaves was performed as described by Chew et al. [4]. 1 g of each sample was frozen with liquid nitrogen and was ground in a mortar. The powdered samples were then extracted with 50 mL 75% v/v methanol and were shook continuously for an hour at room temperature. The extracts were filtered under vacuum and kept at -20°C until further analysis.

#### Antibacterial activity and phytochemical screening

25 g of fresh plant materials were collected and dried for 1 day using freeze dryer. The dried samples were ground into fine powder using household blender. The powdered materials were extracted with 500 mL of dichloromethane:methanol (1:1) mixture with continuous stirring for 3 days at room temperature. Then, the extracts were vacuum filtered, and the plant residues were extracted again with 500 mL of methanol for 1 day at room temperature, and the extracts were filtered again. Both extracts were combined and evaporated under vacuum. The crude extracts were stored in dark at -20°C until further analysis.

### Total phenolic content (TPC)

The total phenolic content (TPC) of extracts was measured using Folin-Ciocalteu method as described previously [4]. 1.5 mL Folin-Ciocalteu's phenol reagent (Sigma) (10% v/v) and 1.2 mL 7.5% w/v Na_2_CO_3 _were added to 0.3 mL sample extract. The reaction mixture was thoroughly mixed and was incubated in the dark for 30 minutes before the absorbance was measured at 765 nm. TPC was expressed in terms of mg gallic acid equivalents (GAE) per 100 g fresh material. The calibration equation for gallic acid was y = 0.0111*x *- 0.0148 (R^2 ^= 0.9998), where *x *is the gallic acid concentration in mg/L and y is the absorbance reading at 765 nm.

### 2, 2-diphenyl-1-picrylhydrazyl (DPPH) radical scavenging assay

DPPH radical scavenging assay was measured according to Chew et al. [4]. 1 mL of extract with various dilutions was added to 2 mL of DPPH (Sigma) (0.15 mM in methanol). The reaction mixtures were incubated for 30 minutes and the absorbance measured at 517 nm. The DPPH scavenging ability was calculated as IC_50 _and expressed in mg ascorbic acid (AA) equivalents per 100 g of fresh material (AEAC) as follows:

AEAC (mg AA/100 g) = IC_50(ascorbate)_/IC_50(sample) _× 10^5^

IC_50 (ascorbate) _of 0.00382 mg/mL was used in AEAC calculation.

### Antibacterial activity

#### Bacteria strains

Eight species (Gram positive and Gram negative) of bacteria were used. The Gram positive bacteria were *Bacillus cereus *ATCC 14579, *Micrococcus luteus *ATCC 4698, methicillin-sensitive *Staphylococcus aureus *(MSSA) ATCC 25923, and two strains of methicillin-resistant *S. aureus *(MRSA) (ATCC 33591 and clinical strain). The Gram negative bacteria include *Escherichia coli *ATCC 25922, *Klebsiella pneumoniae *ATCC 10031, *Pseudomonas aeruginosa *ATCC 10145, and *Enterobacter aerogenes *(obtained from Institute for Medical Research Malaysia). All bacteria were cultured on nutrient agar (Oxoid).

#### Preparation of inoculums

One single colony of each type of microorganism (from the nutrient agar stock culture) was inoculated with a sterile loop, and was transferred into 10 mL sterile nutrient broth (Oxoid). The broth cultures were incubated in a shaking incubator at 37°C for 16 - 20 hours.

#### Antibacterial susceptibility test: Disc diffusion assay

The antibacterial assay was performed using disc diffusion (Kirby-Bauer) method [1]. The density of bacteria was standardized using McFarland 0.5 turbidity standard to 1 × 10^8 ^coliform units (cfu)/mL and was swabbed onto Mueller Hinton Agar (Oxoid) surface. 1 mg of crude extract was dissolved initially in 100 μL methanol and loaded onto sterile Whatman No. 1 filter paper discs (6 mm diameter) and the discs were impregnated onto inoculated agar. 10 μg of streptomycin or 30 μg vancomycin (both from Oxoid) and blank disc without extract were served as positive and negative controls, respectively. The plates were left at 4°C for an hour to allow the diffusion of extracts before they were incubated for 16 - 20 hours at 37°C. Antibacterial activity was indicated when clear inhibition zones were noted around the discs. The diameter of the inhibition zones was measured and the results were expressed as mean of three independent experiments. The test was repeated three times.

#### Determination of minimum inhibitory dosage (MID)

Minimum inhibitory dosage (MID), minimum dose per disc required to inhibit growth of microorganism was performed on extracts which showed positive activity in the preliminary screening. MID was determined as described by Habsah et al. [14]. Serial dilutions of extracts from 0.01 - 1 mg per disc were loaded onto filter paper discs according to disc diffusion method described above.

### Phytochemical analysis

Phytochemical screening for flavonoids, tannins, saponins and alkaloids was determined as described by Parekh and Chanda [15] and Aiyegoro and Okoh [16], but the amount of extract used was increased for better results visualisation. 1 g of extract was dissolved in 10 mL dH_2_O and was then filtered. 10 mg magnesium turnings were added into 1 mL of the filtrate, followed by the addition of 0.05 mL concentrated sulphuric acid. The presence of magenta red observed within three minutes confirmed the presence of flavonoids. The presence of blue-black precipitates resulting from the addition of ferric chloride (0.01 g/mL) reagent indicated the presence of tannins. Frothing test was used to determine the presence of saponins, where 2 mL of the extract was shook vigorously for 4 minutes and the presence of honey-comb froth indicated the existence of saponins. The presence of alkaloids was determined by first dissolving 0.02 g of extract in 1 mL methanol and filtered, followed by boiling the extract with 2 mL of 1% hydrochloric acid for 5 minutes. 4 - 6 drops of Dragendorff's reagent was then added into the extract, and the formation of orange precipitates indicated the presence of alkaloids. Screening the presence of steroids and terpenoids was performed as described by Kumar *et al. *[17]. 0.2 g of extract was dissolved in 10 mL methanol and filtered. 1 mL of chloroform and 1 mL of concentrated sulphuric acid were then added into 1 mL filtrate by the side of the tube and the presence of yellow with green fluorescence at the sulphuric acid layer indicated the presence of steroids. For the detection of terpenoids, 1 mL of acetic anhydride and 2 mL concentrated sulphuric acid were added into 1 mL filtrate. Presence of reddish brown on interface indicated the presence of terpenoids.

### Statistical analysis

All measurements were carried out in triplicate. Statistical analyses were performed using a one-way analysis of variance ANOVA, and the significant difference between means was determined by Duncan's multiple range test. Differences at *P *< 0.05 were considered statistically significant. The results were presented as mean values ± SD (standard deviations).

## Results

### TPC and DPPH scavenging activity

TPC of flowers can be ranked as follows using Folin Ciocalteu assay: *B. kockiana *>*C. surattensis *>*C. pulcherrima *>*A. auriculiformis *≥ *C. tergemina *>*P. pterocarpum *≥ *L. leucocephala *>*S. saman *>*B. purpurea*. Slightly different ranking was observed in TPC of the leaves: *C. pulcherrima *has the highest TPC (5030 ± 602 mg GAE/100 g), followed by *P. pterocarpum *(4880 ± 275 mg GAE/100 g), *B. kockiana *(4220 ± 104 mg GAE/100 g), *C. tergemina *(4200 ± 292 mg GAE/100 g), *C. surattensis *(3330 ± 309 mg GAE/100 g), *A. auriculiformis *(2280 ± 294 mg GAE/100 g), *L. leucocephala *(1700 ± 277 mg/GAE/100 g), *S. saman *(1340 ± 22 mg GAE/100 g) and *B. purpurea *(1310 ± 124 mg GAE/100 g). Statistical analysis showed no significant difference between *C. pulcherrima *and *P. pterocarpum*, *B. kockiana *and *C. tergemina*, *A. auriculiformis *and *L. leucocephala*, *S. saman *and *B. purpurea*. It was observed that *L. leucocephala*, *S. saman *and *B. purpurea *flowers and leaves were ranked similar, with lowest TPC.

**Figure 1:**
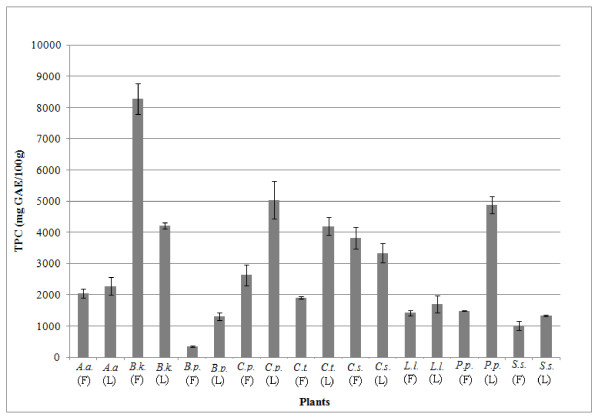
**Total phenolic content (TPC) of flowers (F) and leaves (L) of Leguminosae species**. Results are expressed as means ± SD (n = 3). *A.a. *= *A. auriculiformis*; *B.k*. = *B. kockiana*; *B.p. *= *B. purpurea*; *C.p. *= *C. pulcherrima*; *C.t. *= *C. tergemina; C.s*. = *C. surattensis*; *L.l. *= *L. leucocephala*; *P.p. *= *P. pterocarpum*; *S.s. *= *S. saman*.

It was found that free radical scavenging activity of the plants was directly proportional to TPC (Figure 1 and Table 1), whereby the higher the TPC of the extract, the stronger the free radical scavenging activity. *B. kockiana *flower displayed the highest TPC (8280 ± 498 mg GAE/100 g) and AEAC (14 600 ± 2360 mg AA/100 g) and strongest DPPH radical quenching ability, in which 27.0 ± 5.0 μg/mL of extract could effectively scavenge 50% of the free DPPH radicals. Whereas, *B. purpurea *flower consists of much lower TPC and AEAC (349 ± 26 mg GAE/100 g and 235 ± 35 mg AA/100 g respectively), and higher concentration of extract was needed to scavenge 50% of the free radicals (1690 ± 280 mg/mL). Strong positive association was observed between TPC and AEAC (R^2 ^= 0.9747), while TPC and IC_50 _were inversely proportional (R^2 ^= 0.9753). This shows that radical scavenging activity is closely associated with the phenolic compounds in these plants.

**Table 1:** Free radical scavenging activity of flowers (F) and leaves (L) of Leguminosae species

Plants	Part	Antioxidant activity	
		
		IC_50_(μg/mL)	AEAC (mg AA/100 g)
*A. auriculiformis*	F	152 ± 13^a^	2540 ± 221^a^
	L	161 ± 30^a^	2430 ± 457^a^
*B. kockiana*	F	27.0 ± 5.0^a^	14 600 ± 2360^a^
	L	61.0 ± 10.0^b^	6410 ± 985^b^
*B. purpurea*	F	1690 ± 280^a^	235 ± 35^a^
	L	384 ± 45^b^	1010 ± 122^b^
*C. pulcherrima*	F	118 ± 24^a^	3350 ± 618^a^
	L	50.0 ± 5.0^b^	7690 ± 735^b^
*C. tergemina*	F	179 ± 12^a^	2170 ± 130^a^
	L	61.0 ± 6.0^b^	6300 ± 610^b^
*C. surattensis*	F	96.0 ± 16.0^a^	4080 ± 728^a^
	L	94.0 ± 11.0^a^	4130 ± 463^a^
*L. leucocephala*	F	289 ± 17^a^	1340 ± 78^a^
	L	187 ± 15^b^	2070 ± 156^b^
*P. pterocarpum*	F	189 ± 10^a^	2040 ± 120^a^
	L	51.0 ± 0.0^b^	7630 ± 331^b^
*S. saman*	F	343 ± 43^a^	1130 ± 153^a^
	L	384 ± 39^a^	1000 ± 97^a^

Results are expressed as means ± SD (n = 3). For each column, values followed by the same letter (a-b) are statistically insignificant (*P *> 0.05) as determined using ANOVA, and this does not apply for different plants.

Statistical analysis showed that TPC and antioxidant activity of flowers and leaves of the same species are significantly different. However, exceptions were seen in *A. auriculiformis*, *C. surattensis, L. leucocephala *and *S. saman*. On the other hand, TPC of both parts of *S. saman *were shown to be statistically significant but they were not significant in terms of antioxidant activity.

### Antibacterial activity and minimum inhibitory dosage (MID) of extracts

The susceptibility of bacteria towards the plant extracts was assessed. All nine extracts were tested on selected Gram positive and negative bacteria. The screening showed most extracts displayed a broad spectrum of antibacterial activity towards Gram positive but some had selective activity towards the bacteria, i.e. *B. purpurea *flower exhibited inhibition activity towards antibiotic resistant *S. aureus *(MRSA), but not to the other three Gram positive bacteria (Table 2). No inhibition was observed in Gram negative bacteria at the same dosage for all the plant extracts.

**Table 2:** Antibacterial activity of flowers (F) and leaves (L) of Leguminosae species^a ^evaluated using disc diffusion assay at 1 mg per disc

		Bacterial strains				
		
		Diameter of zone of inhibition (mm)				
		
Plants	Part	*B. cereus *ATCC 14579	*M. luteus *ATCC 4698	MSSA ATCC 25923	MRSA ATCC 33591	MRSA (clinical isolate)^b^
*A. auriculiformis*	F	10.0 ± 0.0	11.8 ± 0.4	11.8 ± 0.4	15.3 ± 0.4	13.3 ± 0.4
	L	9.50 ± 0.7	12.0 ± 0.0	12.0 ± 0.0	15.3 ± 1.1	13.5 ± 0.7
*B. kockiana*	F	10.0 ± 0.0	12.0 ± 0.0	12.5 ±0.7	11.0 ± 0.0	11.5 ± 0.7
	L	8.00 ±0.00	10.0 ± 0.0	9.50 ± 0.70	9.00 ± 0.00	10.0 ± 0.0
*B. purpurea*	F	-	-	-	12.3 ± 0.4	10.0 ± 0.0
	L	7.00 ± 0.00	8.30 ± 0.35	7.00 ± 0.00	8.00 ± 0.00	-
*C. pulcherrima*	F	7.80 ± 0.40	10.0 ± 0.0	10.0 ± 0.0	9.50 ± 0.70	9.50 ± 0.70
	L	7.80 ± 0.40	10.0 ± 0.0	10.0 ± 0.0	10.5 ± 0.7	9.50 ± 0.70
*C. tergemina*	F	7.00 ± 0.00	7.50 ± 0.70	8.50 ± 0.00	9.30 ± 0.35	8.50 ± 0.70
	L	10.3 ± 0.4	11.0 ± 0.0	12.0 ± 0.0	13.0 ± 0.0	11.0 ± 0.0
*C. surattensis*	F	7.50 ± 0.70	10.0 ± 0.0	10.0 ± 0.0	11.0 ± 0.0	10.0 ± 0.0
	L	7.50 ± 0.70	10.0 ± 0.0	10.0 ± 0.0	10.0 ± 0.0	10.0 ± 0.0
*L. leucocephala*	F	-	9.30 ± 0.40	8.00 ± 0.00	11.0 ± 0.0	8.80 ± 1.10
	L	-	9.00 ± 0.00	9.30 ± 0.40	9.30 ± 0.40	10.0 ± 0.0
*P. pterocarpum*	F	8.00 ± 0.0	11.3 ± 0.4	9.80 ± 0.40	10.8 ± 0.4	10.3 ± 0.4
	L	9.80 ± 0.40	12.0 ± 0.0	11.5 ± 0.7	11.8 ± 0.4	10.8 ± 1.1
*S. saman*	F	-	-	-	-	-
	L	-	21.3 ± 0.4	8.50 ± 0.70	9.00 ± 0.00	9.30 ± 0.35
Streptomycin (10 μg/disc)		24.0 ± 0.0	27.0 ± 0.0	14.0 ± 0.0	n.t	n.t
Vancomycin (30 μg/disc)		n.t	n.t	n.t	21.0 ± 0.0	21.0 ± 0.0

Note: diameter of the antibacterial disc is 6 mm. Results are expressed as means ± SD obtained from 3 independent experiments. ^a^All extracts were inactive against Gram negative bacteria (*Escherichia coli *ATCC 25922, *Klebsiella pneumoniae *ATCC 10031, *Pseudomonas aeruginosa *ATCC 10145, *Enterobacter aerogenes *obtained from Institute for Medical Research Malaysia) tested. ^b^Obtained from Faculty of Medicine and Health Sciences UPM. "-" indicates no inhibition activity. "n.t" represents not tested.

Minimum inhibitory dosage (MID) was evaluated to assess the potency of the extracts in inhibiting the growth of bacteria. The antibacterial activity of *B. kockiana *flower and *C. surattensis *leaf extracts were strong. Most of the antibacterial activity of the other extracts exhibited medium to weak inhibition activity. It is interesting to observe that *B. purpurea *flower exhibited medium inhibition activity towards MRSA, but inactive against the antibiotic sensitive strain (MSSA) and other Gram positive bacteria. Besides, it was noted that *S. saman *flower did not exhibit any antibacterial activities towards both Gram positive and negative bacteria tested in this study.

### Phytochemical screening

It has been reported that biological activities in the selected plants were exhibited by different class of phytochemicals [18-24]. Therefore, it is important to screen for phytochemical group in these plants. Phytochemical screening on the 18 extracts showed the presence of different types of chemical constituents. Tannin was detected in most of the extracts (14 extracts), followed by terpenoid (13 extracts), steroid (13 extracts), flavonoid (12 extracts), and saponin (10 extracts). Alkaloid was only present in 4 extracts (Table 3).

**Table 3:** Classes of phytochemicals present in the plant extracts

Plants	Part	Tannin	Terpenoid	Alkaloid	Steroid	Saponin	Flavonoid
*A. auriculiformis*	F	-	+	+	-	+	-
	L	-	+	-	+	+	-
*B. kockiana*	F	+	-	-	+	-	+
	L	+	-	-	+	-	+
*B. purpurea*	F	-	+	+	+	+	+
	L	-	+	-	+	+	-
*C. pulcherrima*	F	+	+	-	+	-	+
	L	+	+	-	+	-	+
*C. tergemina*	F	+	+	-	+	+	+
	L	+	+	-	+	+	+
*C. surattensis*	F	+	+	-	-	-	+
	L	+	-	-	-	-	+
*L. leucocephala*	F	+	-	+	-	-	+
	L	+	-	-	+	+	+
*P. pterocarpum*	F	+	+	-	-	+	-
	L	+	+	-	+	+	-
*S. saman*	F	+	+	+	+	+	+
	L	+	+	-	+	-	-

Note: "+" = present; "-" = absent

## Discussion

### Antioxidant activity

Plants produce diverse arrays of phytochemicals which are useful in the development of new drugs. These phytochemicals are mostly secondary metabolites constantly synthesized by the plant for defensive purposes [4]. For instance, antioxidants are biologically produced as defensive mechanism to prevent tissues destruction caused by highly reactive chemical species, which are formed from various biochemical reactions.

Excessive free radicals which are produced from various biochemical reactions such as triplet chlorophyll, singlet oxygen and hydroxyl radicals are lethal to plants. One of the major classes of natural antioxidants found in plants that remove such free radicals is polyphenols. Polyphenols are able to neutralize free radicals, scavenge singlet and triplet oxygen, and to break down peroxides. Total polyphenol content (TPC) of plants was evaluated using Folin-Ciocalteu method, which measured the redox properties of polyphenols in extracts [4].

The amount of TPC of selected plants could be categorised into 4 classes: high (>5000 mg GAE/100 g), medium high (3000 - 5000 mg GAE/100 g), medium low (1000 - 3000 mg GAE/100 g) and low (<1000 mg GAE/100 g). According to Kuete and Efferth [25], free radical scavenging ability was also ranked according to IC_50_: high (<50 μg/mL); moderate (50 - 100 μg/mL) and low (>50 μg/mL). Most of the extracts showed medium level of TPC (medium high: 5 extracts; medium low: 10 extracts). It was noticed that most leaves had higher TPC than the flowers of the same species, i.e. TPC of *B. purpurea*, *C. pulcherrima*, *C. tergemina*, *P. pterocarpum *and *S. saman *leaves were significantly higher than their respective flowers (Figure 1). Although TPC of *C. surattensis *flower was found to be higher than its leaves, they were statistically not significant. From this interesting observation, it could be explained by the differences in antioxidant distribution in the leaves and flowers. Similar observation was discovered in *Cassia fistula *[26] and *Cynara cardunculus *[27]. Siddhuraju et al. [26] reported the production of anthraquinones, xanthones, flavonol and proanthocyanidins in leaves would lead to higher antioxidant activity. In addition, del Bano et al. [28] found that some antioxidative compounds were selectively biosynthesized by the leaves but the compounds do not exist in flowers. Our previous study [4] also showed that rutin and chlorogenic acid were present in *B. kockiana *and *C. surattensis *leaves but they were absent in their respective flowers. This may be due to higher stress level experienced by the leaves in the process of photosynthesis, where excessive light energy may be absorbed by the leaf tissues. The chlorophylls would also undergo photosensitization process (process of transferring absorbed light energy), which may trigger the production of highly reactive chemical species at cellular level [29]. Therefore, leaf tissues would need to generate highly effective antioxidants and free radical scavengers to aid in quenching and removing the ROS and to minimize the photosensitization-induced oxidative damage.

Compared to the results showed in most plants, exception was seen in *B. kockiana*. *B. kockiana *showed a very unique trend of TPC and free radical quenching ability distribution in the leaves and flowers among the nine selected plants. The TPC and free radical quenching ability were significantly higher in flowers than leaves. Our earlier report [4] showed the presence of high anthocyanins content in *B. kockiana *flowers. Anthocyanins which act as visual attractant for potential pollinators (i.e. insects) may have contributed to the high phenolic content and strong free radical scavenging power of *B. kockiana *flower extract. Anthocyanins are the common pigments in coloured flowers that could terminate radical chain reaction, prevent both enzymatic and non-enzymatic lipid peroxidation of cell membranes by binding with fatty radicals [4].

An antioxidative xanthophylls and lutein were found in *C. surattensis *flower [4]. Lutein protects photo-induced free radical damage in plant tissues in 2 ways: filters off the high energy blue light and scavenges the reactive chemical species [30]. The presence of rutin (95.7 mg/100 g) and chlorogenic acid (9.13 mg/100 g) in extract would exhibit antioxidant activities. Our previous study [4] has shown that rutin was present in *C. surattensis *flowers and leaves extracts (13.3 mg rutin equivalent/100 g and 29.6 mg rutin equivalent/100 g, respectively), assessed using aluminium chloride method. Rutin (95.7 mg/100 g) and chlorogenic acid (9.13 mg/100 g) in extract would exhibit antioxidant activities [4].

### Antibacterial activity and phytochemicals screening

All extracts were not active against Gram negative bacteria. This could be due to the presence of outer membrane as a permeability barrier in Gram negative bacteria. The presence of porin at the outer membrane of Gram negative bacteria restricted the diffusion of many antibiotics and the multidrug efflux pumps at the transmembrane would also pump out the antibacterial agents through the active efflux processes which would hence create a higher intrinsic resistance for Gram negative bacteria [31]. It is interesting to note that this is the first report on the inhibition activity of most plant extracts against MRSA.

As phytochemicals often play an important role in plant defence against prey, microorganism, stress as well as interspecies protections, these plant components have been used as drugs for millennia. Hence, phytochemicals screening serves as the initial step in predicting the types of potential active compounds from plants.

The presences of flavonoids [32], triterpenoid saponins [33] and tannins [34] in *A. auriculiformis *have been reported. Good DPPH free radical scavenging activity of *A. auriculiformis *is not only attributed to the presence of tannins, but also leucoanthocyanidins (leucodelphinidins and leucocyanidins) which are present abundantly in the plant. This is because of the hydroxylation at 4' orthoposition of leucoanthocyanidins that could change DPPH radical to non-radical DPPH-H [35]. Besides, two acylated bisglycoside saponins, acaciaside A and B isolated from *A. auriculiformis*, were found to exhibit antibacterial and antifungal activity. Mandal et al. [36] found that mixture of these two saponins are able to inhibit conidial germination of *Aspergillus ochraceous *and *Curvularia lunata *at 300 μg/mL while the bactericidal concentration of the mixture against *Bacillus megaterium*, *Salmonella typhimurium *and *P. aeruginosa *was at 700 μg/mL or higher. Also, three antifungal flavonoids were found in heartwood of *A. auriculiformis*. Mihara et al. [18] proposed that the antifungal activity of 3,4',7,8-tetrahydroxyflavone and teracacidin were correlated to the antioxidant activities, where these antifungal flavonoids could scavenge the free radicals produced by extracellular fungal enzyme laccase, hence inhibiting the mycelia growth of fungus.

The extract of *C. pulcherrima *has been reported to inhibit the growth of a broad spectrum of bacteria [19,37,38]. The extract has also shown antifungal activity against plant pathogens and yeast [37,38]. However, none of the Gram negative bacteria were inhibited by *C. pulcherrima *extracts in our preliminary screening. Mukherjee and Ray [39] and Ali et al. [19] reported the presence of all five classes of phytochemicals in the leaves while tannins, saponins and alkaloids were detected in the aerial parts [15]. In contrary, our findings showed alkaloid and saponin were not detected in this plant. The presence of gallic acid, ellagic acid and flavonoids (i.e. myricetin, catechin, rutin, and quercetin) were found in *C. pulcherrima *flowers [40,41]. However, our previous report [4] showed the presence of rutin but absence of tannins and other flavonoids. This can be explained by the ecophysiological effect and abiotic growth factors that would possibly be the major determining factors which could modify the expression of phytochemicals in the plant [4]. Terpenoids were found in *C. pulcherrima *[20,21,42,43]. Phytochemical compounds in *C. pulcherrima *that possessed antimicrobial abilities have been isolated. For instance, Srinivas et al. [22] discovered 5,7-dimethylenedioxyflavanone, isobouducellin and 2'hydroxy-2,3,4',6'-tetramethoxychalcone that possessed moderate to good antimicrobial activity against *S. aureus*, *B. subtilis*, *B. sphaericus*, *A. niger*, *R. oryzae*; 3-oxo-(20S,24S)-epoxydammarane-19,25-diacetate from *C. pulcherrima *bark. It has exhibited moderately strong antibacterial activity towards *B. cereus *(MIC 16 μg/mL) and *Shiegella dysenteriae *(MIC 32 μg/mL) [20]; and several cassane-furanoditerpenoids were found to exhibit fairly strong inhibition activity against various bacteria and fungus [42,43].

Positive results were seen in *L. leucocephala *leaves in alkaloid screening (Table 3). It has been reported that mimosine, an alkaloid was detected in this plant [23,44]. Studies found that 5.35 g of mimosine were present in 100 g of dry *L. leucocephala *leaves, 0.53 g/100 g in nodules, 1.49 g/100 g in roots [44] and 2.38 g/100 g in mature seeds [45], but no reports found the presence of mimosine in the flowers, and our studies confirmed the absence of alkaloid in the flowers. Chanwitheesuk et al. [46] reported the presence of tannins, vitamin E, ascorbic acid, carotenes, xanthophylls and phenolics in *L. leucocephala *and they were known to be antioxidative substances, but may also possessed antibacterial properties.

Duraipandiyan et al. [47] reported that methanolic extract of *P. pterocarpum *flower displayed moderate antimicrobial activity against several bacteria strains. In addition, fairly strong antifungal activity (>50% inhibition) of leaves extract against *Fusarium *sp. [48], *Aspergillus *sp. [24] and *Cladosporium cucumerinum *[49] were reported, and this shows that *P. pterocarpum *leaf has antifungal bioactive agents which could be applied as fungicide.

This is the first antioxidant, antibacterial and phytochemical screening study for *S. saman *flowers. The presence of low tannin and flavonoid contents in flower could have been contributed to medium low TPC and free radical scavenging activity (Figure 1 and Table 1). However, it was found that the flower extract showed no antibacterial activity (Table 2), and this showed that the phytochemicals present in the flower may not possess any inhibiting activity against the eight bacteria. Our study found the presence of tannins, terpenoids and steroids in the *S. saman *leaves, and the results were consistent with reported findings [50]. However, the presence of saponins and flavonoids in leaves was in contrary to our findings [50]. Mild to medium inhibition antimicrobial activity of *S. saman *leaves extract against various Gram negative bacteria was reported [50]. Ali et al. [19] discovered that antibacterial activity of methanolic extract of *S. saman *leaves was significantly stronger than non-polar hexane extract and this showed that the antibacterial agents in the leaves were mostly hydrophilic in nature. In addition, it was discovered that alkaloid fractions exhibited fairly strong antibacterial activity (MIC values ranging between 7 μg/mL and 20 μg/mL) [50]. The antibacterial activity of *S. saman *leaves could also be exhibited by alkaloids detected in the extracts, as alkaloid fractions of *S. saman *was reported to be able to inhibit growth of various bacteria and it was equally potent as gentamycin [50].

## Conclusions

In conclusion, our findings showed some Leguminosae plants, such as *B. kockiana*, *C. pulcherrima*, *C. tergemina *and *P. pterocarpum *have the potential to be explored further to identify the antioxidative and antibacterial compounds in these plants. The present results will serve as the preliminary findings for selection of potential plant species for further investigation, especially in isolating new bioactive compounds, which have strong antioxidant activity and anti-MRSA ability. Studies on isolating bioactive compounds using bioassay guided approach are in progress.

## Competing interests

The authors declare that they have no competing interests.

## Authors' contributions

YLC, EWLC and PLT performed the experimentation as part of their PhD and Honours studies. YLC and EWLC prepared the manuscript. JKG, YYL and JS supervised the work, evaluated the data and corrected the manuscript for publication. All authors read and approved the final manuscript.

## Pre-publication history

The pre-publication history for this paper can be accessed here:

http://www.biomedcentral.com/1472-6882/11/12/prepub
